# Long-term prophylaxis in an immunocompetent patient with Cytomegalovirus retinitis: a case report and review of literature

**DOI:** 10.1186/s12348-020-00207-1

**Published:** 2020-06-26

**Authors:** Seyed Ali Tabatabaei, Kasra Cheraqpour, Elias Khalili Pour, Bahram Bohrani Sefidan

**Affiliations:** Eye Research Center, Farabi Eye Hospital, Tehran University of Medical Sciences, Tehran, Iran

**Keywords:** Cytomegalovirus retinitis, Posterior uveitis, CMV, CMV retinitis in immucocompetent patients, Prevention and control

## Abstract

**Background:**

Cytomegalovirus retinitis is an infectious sight-threatening condition that usually occurs in immunosuppressed individuals, but rare cases of Cytomegalovirus retinitis have been reported in immunocompetent patients.

**Findings:**

A 68-year-old woman without any history of systemic diseases referred to the emergency ward of Farabi eye hospital with a two-week history of decreased vision in her left eye. Fundoscopy of the left eye revealed mild venous tortuosity, hemorrhagic retinitis within the macula, and papillitis. The right eye had a history of Cytomegalovirus retinitis 2 years ago that complicated with rhegmatogenous retinal detachment. Immunologic evaluations were normal without any sign of immunosuppressive conditions. She was treated with intravenous ganciclovir for 2 weeks, intravitreal ganciclovir (twice weekly) for 1 week, and also daily oral valganciclovir as maintenance therapy for 6 months resulted in resolving of retinitis patches and improving her best-visual acuity from hand motions to 20/100. Forty-five days after stopping maintenance therapy recurrence occurred. So we started the treatment again to stabilize the patient. She is currently maintained on valganciclovir 900 mg daily without recurrence for 9 months.

**Conclusions:**

Cytomegalovirus retinitis can recur in the same or contralateral eye of immunocompetent patients, especially without prophylactic medication.

## Introduction

Cytomegalovirus retinitis (CMVR) is a sight-threatening condition usually affecting immunosuppressed individuals but few cases of CMVR have been reported in immunocompetent patients [1]. Herein, we report an immunocompetent patient with unsynchronized bilateral involvement without a previous predisposing factor for acquiring CMVR.

## Case presentation

**Fig. 1:**
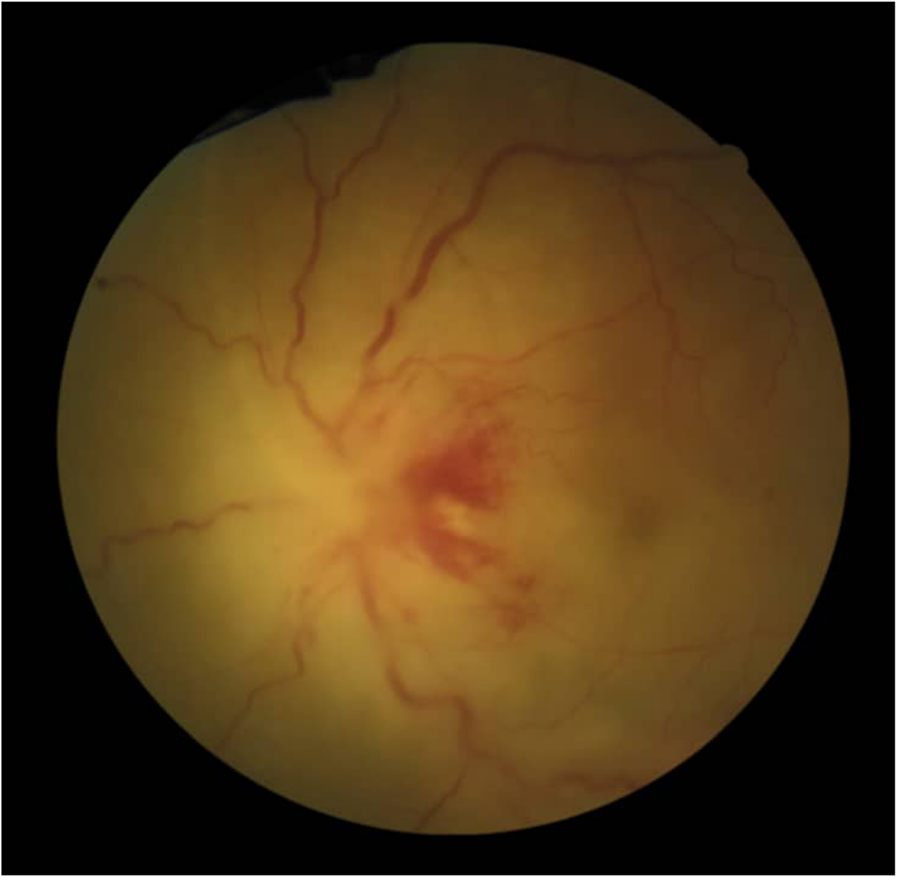
Fundoscopy of the left eye: mild venous tortuosity, hemorrhagic retinitis within the macula, and papillitis

**Fig. 2:**
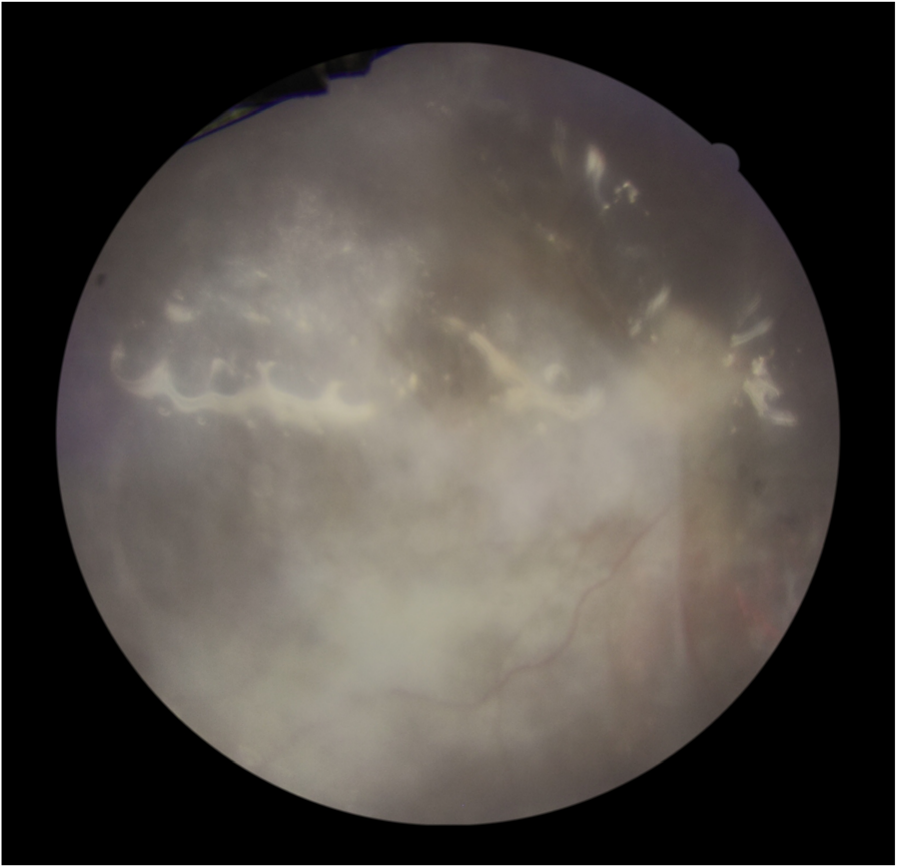
Fundoscopy of the right eye: Silicone oil-filled vitreous, pale optic disk, occluded retinal vessels, and diffuse chorioretinal atrophy

A 68-year-old woman without any history of systemic diseases was referred to the emergency ward of Farabi eye hospital with a two-week history of decreased vision in her left eye. At presentation, her best-corrected visual acuity (BCVA) was hand motions in her left eye and no light perception in the right eye. On the slit-lamp examination, the left eye had fine diffuse keratic precipitates and 1+ anterior chamber cells. Also, fundoscopy revealed mild venous tortuosity, hemorrhagic retinitis within the macula, and papillitis (Fig. 1). The first episode of CMVR has been occurred in the right eye about 2 years ago which complicated with the rhegmatogenous retinal detachment (RRD) after 3 months treatment with valganciclovir and underwent pars planavitrectomy with silicone oil injection. Current fundus examination of the right eye revealed pale optic disc, occluded retinal vessels, and diffuse chorioretinal atrophy (Fig. 2).

The referring ophthalmologist confirmed the diagnosis of CMVR in the right eye after vitreous sampling and CMV PCR assessment. The patient had close follow up visits and immunologic status including complete blood cell count and lymphocytes count have been checked out frequently without any sign of immunosuppression. Upon initiating symptoms in the left eye, the patient was referred to our center for more assessments.

Due to unusual presentations of patient, infectious and hematologic consultations and vitreous sampling were scheduled. The requested laboratory tests including complete blood count (CBC), Erythrocyte Sedimentation Rate (ESR), C-Reactive Protein, absolute count of lymphocytes, CD3+, CD4+ (609 cells/μl), CD8+, CD16+ and CD56+ lymphocyte count, complement system function, autoimmune antibodies like Anti – Neutrophil Cytoplasmic Antibody (C-ANCA, P-ANCA), Anti-Nuclear Antibody (ANA), and Rheumatoid Factor (RF), Veneral Disease Research Laboratory (VDRL), Fluorescent Treponemal Antibody Absorption (FTA-ABS), liver function tests, creatinine, Fasting Blood Sugar (FBS), Purified Protein Derivative (PPD), Hepatitis B virus antigen (HBs Ag), Hepatitis C virus antibody (HCV Ab), anti-HIV antibody, all were in normal laboratory ranges. DNA PCR of Varicella-zoster Virus (VZV), Herpes Simplex Virus (HSV), and CMV on either whole blood or vitreous samples were negative except positive CMV DNA PCR of the vitreous sample. Also, requested consultations did not reveal any underlying immunodeficiency and malignancy evaluation was negative.

**Fig. 3:**
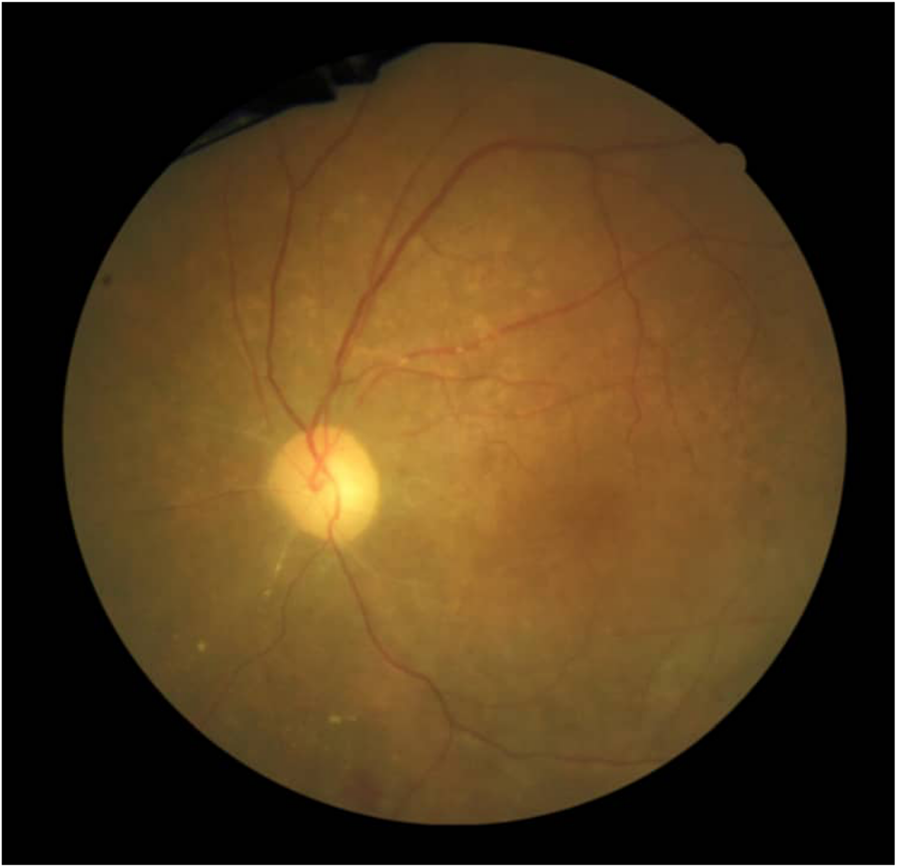
Fundoscopy of the left eye after the treatment

**Fig. 4:**
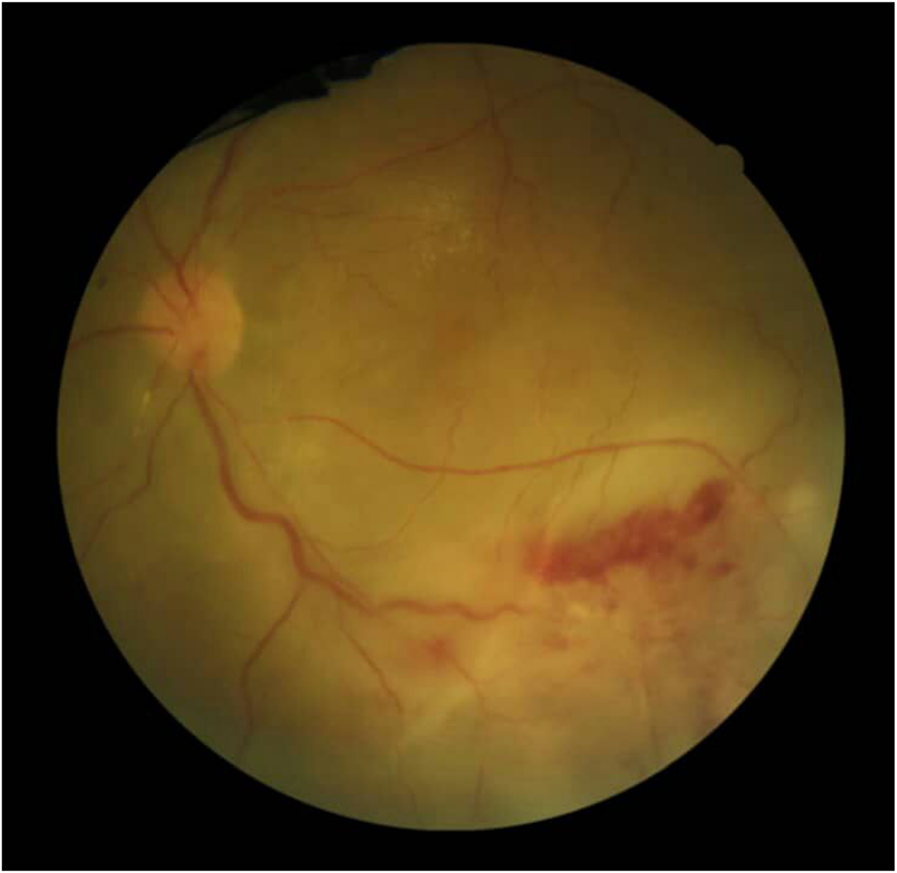
Fundoscopy of the left eye, recurrence after stopping maintenance therapy

Because of clinical features and previous history of CMVR, treatment has been started against CMV. The devastating course of the disease in the fellow eye and involvement of the posterior pole and optic disk persuasive us for aggressive treatment. So we started the treatment with intravenous ganciclovir 10 mg/kg/day for 2 weeks and 2 mg injections of intravitreal ganciclovir (twice weekly) for 1 week. The treatment followed by 900 mg daily oral valganciclovir as maintenance therapy for 6 months. During the treatment, her visual acuity improved from hand motions to 20/100, and patches of retinitis start to fade from the macula (Fig. 3). Forty-five days after stopping maintenance therapy with valganciclovir retinitis recurred in the left eye and visual acuity dropped to counting fingers at 3 m (Fig. 4), so we started the treatment again to stabilize the patient. She is currently maintained on valganciclovir 900 mg daily without recurrence for 9 months with 20/100 visual acuity.

## Discussion

CMVR accounts as the most common opportunistic viral infection in HIV patients which often manifests in CD4+ counts lower than 50/μl [1]. Also, the other immunosuppressive conditions like leukemia, lymphoma, and organ transplant surgery may prone the patient to this form of posterior uveitis [1]. Few reports of CMVR in immunocompetent patients can be found in the literature. However, the majority of these cases have been suffered from subclinical immunosuppression such as diabetes mellitus (DM) [2–6].

Moreover, the role of intravitreal injections is considerable; there are several reports of developing CMVR after intravitreal injection of corticosteroids such as triamcinolone acetonide (TA) [2, 3, 5]. Also,Witmer et al. [7] reported this condition after the administration of intravitreal dexamethasone implant in an immunocompetent old woman. The interesting point is available reports of CMVR after intravitreal injection of anti-vascular endothelial growth factors (anti-VEGFs) like bevacizumab [4, 6]. Although the immunosuppressive effects of corticosteroids may justify the occurrence of this condition, anti-VEGF drugs have not this property. We speculated the disruption of the blood-retinal barrier and subclinical immunosuppression by diabetic retinopathy is the main predisposing factor for the occurrence of CMVR in these cases and considering a separate role for intravitreal injections is difficult. Similar to our case, the majority of previous studies have reported the CMVR in old aged immunocompetent patients. Thus, we think old ages may be associated with higher rates of CMVR. However, Hosseini et al. [8] reported CMVR in a 4-month-old healthy infant recently. A brief review of some of the recent reports of CMV retinitis in immunocompetent patients and issues including age, laterality of involvement, risk factors, manifestations, treatment, and the visual outcome are summarized in Table 1.

**Table 1:** A brief review of some of the recent reports of CMV retinitis in immunocompetent patients. (Abbreviations: Diabetes mellitus (DM), injection of triamcinolone acetonide (IVTA), central retinal vein occlusion (CRVO))

case (Reference)	Age	Laterality	Systemic and ocular history	Manifestation	Treatment	Initial visual acuity	Final visual acuity
1 [1]	61	Right eye	None	Fine keratic precipitates, 1+ aqueous cells, mild vitritis, patches of hemorrhagic retinitis and perivascular sheathing	Intravitreal ganciclovir followed by oral valganciclovir	4/20	8/20
2 [9]	51	Both eyes	None	Fine keratic precipitates, 1+ aqueous cell, and flare, 1+ vitritis in both eyes, areas of confluent retinitis and hemorrhage and peripheral necrosis and fibrosis	Oral valganciclovir	20/60 (right eye) and 20/50 (left eye)	20/70 (right eye) and 20/60 (left eye)
3 [5]	75	Left eye	Diabetes mellitus (DM), intravitreal injection of triamcinolone acetonide (IVTA)	The rare cell in the anterior chamber, no vitreous cells, arcuate retinal whitening (which appeared full-thickness) along the temporal vascular arcades	Intravitreal ganciclovir followed by oral valganciclovir	20/80	20/400
4 [2]	77	Right eye	DM, IVTA	Fine keratic precipitates, 2 + vitritis, foci of oedematous retinitis with dense retinal whitening, hemorrhages and inflammatory retinal vascular sheathing	Intravitreal injection of ganciclovir + intravenous administration of ganciclovir followed by oral valganciclovir	counting fingers at 1.8 m	20/200
5 [2]	69	Right eye	DM, central retinal vein occlusion (CRVO), Vitrectomy surgery, IVTA	Keratic precipitates, 2 + cells in the anterior chamber, moderate vitritis, necrotizing retinitis extending throughout the entire retina with hemorrhages, retinal opacification and periphlebitis	Intravitreal injection of ganciclovir + intravenous administration of ganciclovir followed by oral valganciclovir	20/200	20/400
6 [3]	54	Left eye	Carrier of human T-cell lymphotropic virus type 1, DM, CRVO, vitrectomy surgery, IVTA	Small granulomatous keratic precipitates, 1+ aqueous cell and flare, 1+ vitritis, papillitis, retinal vasculitis, and retinal exudates	Intravenous injections of ganciclovir + Intravitreous injections of foscarnet	1.0	0.5
7 [10]	78	Right eye	IVTA	Keratic precipitates, 2+ cells in the anterior chamber and moderate vitritis, hemorrhagic sheathing of branch arteries, inflammatory vascular sheathings in the periphery and areas of retinal whitening in the peripheral superior nasal part	Intravitreous injections of ganciclovir + intravenous ganciclovir followed by oral valganciclovir	20/40	20/25
8 [4]	61	Left eye	DM, intravitreal injection of bevacizumab	4+ cells and in the anterior chamber, dense vitritis and retinal vascular obliteration, necrotizing retinitis with dense retinal whitening and hemorrhage	Intravenous administration of ganciclovir	Hand motions	Not reported
9 [6]	75	Left eye	DM, intravitreal injection of ranibizumab	Mild anterior segment inflammation, low-grade vitritis, and foci of retinitis in the peripheral fundus along with retinal and subretinal hemorrhages, exudates, and macular edema	Intravitreal ganciclovir	4/10	5/10
10 [11]	74	Right eye	Multiple intraocular surgeries, long-term use of immunosuppressive eye drops such as corticosteroids and cyclosporine	No inflammatory cells in the anterior chamber or the anterior vitreous, confluent exudate the entire peripheral retina	Intravenous ganciclovir+ intravitreal ganciclovir injections followed by oral valganciclovir	Hand motions	Not reported

To the best of the author’s knowledge and database searches like PubMed, few cases of CMVR have been reported in patients without any type of systemic immunosuppression. The physiopathology of developing CMVR in completely immunocompetent patients is not clear. We hypothesized maybe genetic susceptibility acts as a predisposing factor in these cases, similar to the patients with AIDS [12].

CMVR should be differentiated from acute retinal necrosis (ARN), progressive outer retinal necrosis (PORN), toxoplasmosis, and syphilis [13]. History of this form of posterior uveitis, clinical characteristics, and also positive PCR sampling were differentiating points in this case. It is noticeable up to 24% of CMVR cases may lead to RRD as happened in the right eye of the current case we reported [1].

Unsynchronized bilateral involvement is a remarkable aspect of this case. Another unique aspect of this case is recurrence after stopping valganciclovir that debates a challenging question about the duration of prophylaxis in patients without immunosuppression. The answer to this question is not clear and none of the previous reports discussed this issue. Previously, CMVR in an HIV patient indicated life-long anti-CMV therapy. However, development of new treatment options made discontinuation of maintenance therapy safe in these patients due to quantitative immune recovery in low counts of CD4+ lymphocytes as the predisposing factor. According to CDC guidelines maintenance therapy or secondary prophylaxis in AIDS-related CMV retinitis should be continued until reaching to adequate immune recovery which defines as sustained raise of CD4+ cell counts to above 100 cells/μl for at least 3–6 months with inactive CMVR characterized by retinal scarring. These guidelines also mentioned that valganciclovir primary prophylaxis against CMVR is not neccessary once CMVR treatment is successfully completed unless CD4+ count has decreased to < 100 cells/μL [14]. It seems in patients without clear predisposing factor discontinuing of the treatment is not rational and routine ophthalmic monitoring appears useful.

## Conclusion

This report targets bringing issues to light that CMVR can recur in the same or contralateral eye of immunocompetent patients, especially without prophylactic medication.

## Data Availability

The data in the current case report are available from the Farabi Eye Hospital medical records. The data is available from the corresponding author on reasonable request.

## Abbreviations

CMVR: cytomegalovirus retinitis
CMV: cytomegalovirus
RRD: rhegmatogenous retinal detachment
AIDS: acquired immunodeficiency syndrome
VEGF: vascular endothelial growth factor
CD: a cluster of differentiation
PCR: polymerase chain reaction
DM: diabetes mellitus
IVTA: injection of triamcinolone acetonide
CRVO: central retinal vein occlusion

## References

[1] Karkhaneh R, Lashay A, Ahmadraji A (2016). Cytomegalovirus retinitis in an immunocompetent patient: a case report. J Current Ophthalmol.

[2] Delyfer MN, Rougier MB, Hubschman JP, Aouizérate F, Korobelnik JF (2007). Cytomegalovirus retinitis following intravitreal injection of triamcinolone: report of two cases. Acta Ophthalmol Scand.

[3] Furukawa M, Kumagai K, Ogino N, Okinami S, Uemura A, Larson E (2007). Cytomegalovirus retinitis after intravitreous triamcinolone treatment of a vitrectomized eye in an immunocompetent patient. Retinal Cases Brief Reports.

[4] Bae SH, Kim TW, Chung H, Heo JW (2013). Cytomegalovirus retinitis after intravitreal bevacizumab injection in an immunocompetent patient. Korean J Ophthalmol.

[5] Saidel MA, Berreen J, Margolis TP (2005). Cytomegalovirus retinitis after intravitreous triamcinolone in an immunocompetent patient. Am J Ophthalmol.

[6] Zafeiropoulos P, Tamboura P, Dimou M, Christodoulou E, Stefaniotou M (2019). Cytomegalovirus retinitis, in a diabetic immunocompetent patient, after intravitreal ranibizumab injection. Eur J Ophthalmol.

[7] Witmer MT, Connolly BP. Cytomegalovirus retinitis after an intravitreal dexamethasone implant in an immunocompetent patient. Retinal Cases Brief Reports. 2019. 10.1097/ICB.0000000000000904 31339873

[8] Hosseini SM, Moosavi M-N, Shoeibi N, Sakhaee M, Ghavamsaeedi H (2017). Bilateral cytomegalovirus retinitis in a healthy infant. J Current Ophthalmol.

[9] Stewart MW, Bolling JP, Mendez JC (2005). Cytomegalovirus retinitis in an immunocompetent patient. Arch Ophthalmol.

[10] Vertes D, Snyers B, De Potter P (2010). Cytomegalovirus retinitis after low-dose intravitreous triamcinolone acetonide in an immunocompetent patient: a warning for the widespread use of intravitreous corticosteroids. Int Ophthalmol.

[11] Toriyama K, Suzuki T, Hara Y, Ohashi Y (2012). Cytomegalovirus retinitis after multiple ocular surgeries in an immunocompetent patient. Case Reports Ophthalmol.

[12] Sezgin E, Jabs DA, Hendrickson SL, Van Natta M, Zdanov A, Lewis RA (2010). Effect of host genetics on the development of cytomegalovirus retinitis in patients with AIDS. J Infect Dis.

[13] Yeung IY, Downes KM, Cunningham E, Sen HN. CMV retinitis: reduced incidence, still a threat. Review of Ophthalmology May 10th Issue. 2016

[14] Benson CA, Brooks JT, Holmes KK, Kaplan JE, Masur H, Pau A (2009). Guidelines for prevention and treatment opportunistic infections in HIV-infected adults and adolescents; recommendations from CDC, the National Institutes of Health, and the HIV medicine association/Infectious Diseases Society of America.

